# Data on the impact of objects with different shapes, masses, and impact velocities on a dummy head

**DOI:** 10.1016/j.dib.2018.11.143

**Published:** 2018-12-06

**Authors:** Ahmad Yaser Alhaddad, John-John Cabibihan, Ahmad Hayek, Andrea Bonarini

**Affiliations:** a Qatar University, Department of Mechanical and Industrial Engineering, Doha 2713, Qatar; b Politecnico di Milano, Department of Electronics, Information and Bioengineering, Piazza Leonardo da Vinci 32, Milano 20133, Italy

## Abstract

In this article, a data generated from impacts of objects with different shapes, masses, and impact velocities on a developed dummy head. The mass considered was in the range of 0.3–0.5 kg while the shapes considered were cube, wedge, and cylinder. The impact velocities levels were in the range of 1–3 m/s. A total of 144 experiments were conducted and the corresponding videos and raw data were analyzed for impact velocity, peak head linear acceleration, 3 ms criterion, and the Head Injury Criterion (HIC). This dataset includes the raw acceleration data and a summary of the overall processed data. The data is available on Harvard Dataverse: https://doi.org/10.7910/DVN/AVC8GG.

**Specifications table**

**Table:** 

Subject area	*Engineering*
More specific subject area	*Safety*
Type of data	*Dataset and Tables*
How data was acquired	*The data was collected from a developed dummy head embedded with a low-cost triple-axis accelerometer.*
Data format	*Raw and analyzed.*
Experimental factors	*The data was acquired from a dummy head due to the varying of 3 impact factors, namely, mass, shape, and impact velocity.*
Experimental features	*The developed dummy head was subjected to different impacts and the corresponding accelerations (in the X, Y, and Z axes) were stored and then analyzed. Video recordings were used to estimate the impact velocity.*
Data source location	*Qatar University, Doha 2713, Qatar.*
Data accessibility	*The data is available on Harvard Dataverse:*https://doi.org/10.7910/DVN/AVC8GG.
Related research article	A.Y. Alhaddad, J.J. Cabibihan, A. Bonarini, Head impact severity measures for small social robots thrown during meltdown in autism. Int. J. Soc. Robot. (2018) 1–16. https://doi.org/10.1007/s12369-018-0494-3.

**Value of the data**

• The data could be used for safety purposes.
• The data could potentially used to predict the potential of harm to the head due to impacts with small objects.
• The data could be used to help in understanding the dynamics of sub-concussive or concussive events among children.
• Manufacturers of small toys could use such data to optimize their designs.

## 1 Data

The raw data files contain the acceleration readings in the National Instruments (NI) TDMS format^1^. Each file contains columns representing the raw acceleration readings in the X, Y, and Z axes reported in gravitational acceleration (*g* = 9.81 m/s^2^). The processed summary file contains the analysis for three severity indices, namely, peak head linear acceleration in (*g*) unit, 3 ms criterion in (*g*) unit, and Head Injury Criterion.

### 1.1 Detailed description of the data files

The online repository contains raw data files and a summary of the analysis [1]. File ***raw_acceleration_data_labview.rar*** contains the raw data of the 3-axis accelerometer. It is divided to three subfolders based on the shapes used, namely, cube, wedge, and cylinder. Each shape folder is further subdivided to three subfolders based on the range of the mass considered, which were 0.3 kg, 0.4 kg, and 0.5 kg. Finally, each folder of the mass contains 16 files representing 16 different experiments at different impact velocities levels.

The columns and their corresponding data are as follows:

• Column *C (Voltage_2 (Formula Result))* corresponds to the linear acceleration of the dummy head measured in (g) unit.
• Column *D (Voltage_1 (Formula Result))* corresponds to the force sensor readings in Newton (N). This sensor was not used in these experiments.
• Column *E (Voltage_2 (Formula Result)1)* corresponds to the readings of the accelerometer in the X axis in (g) unit.
• Column *F (Formula Result)* corresponds to the readings of the accelerometer in the Y axis in (g) unit.
• Column *G (Formula Result 1)* corresponds to the readings of the accelerometer in the Z axis in (g) unit.

File ***processed_summary.xlsx*** contains a summary of the processed data for the 144 experiments conducted subdivided into tabs corresponding to the different shapes considered. This file contains the analysis for the three severity indices considered.

## 2 Experimental design, materials, and methods

### 2.1 Experimental Setup

A 3D printed head made of polylactide (PLA) was augmented with clay to reach a mass of 3.1 kg. The mass of the dummy head is comparable to that of a scaled down 50^th^ percentile adult head that is used in children dummy heads. A 2 mm layer of deformable soft material made of silicone (Ecoflex 00–30, Smooth-On, USA) was added to the head form to add more lifelike skin [2]. A tri-axial accelerometer (ADXL377, SparkFun Electronics, Colorado, USA) was placed at the center of the head to measure the linear acceleration of the head.

**Fig. 1:**
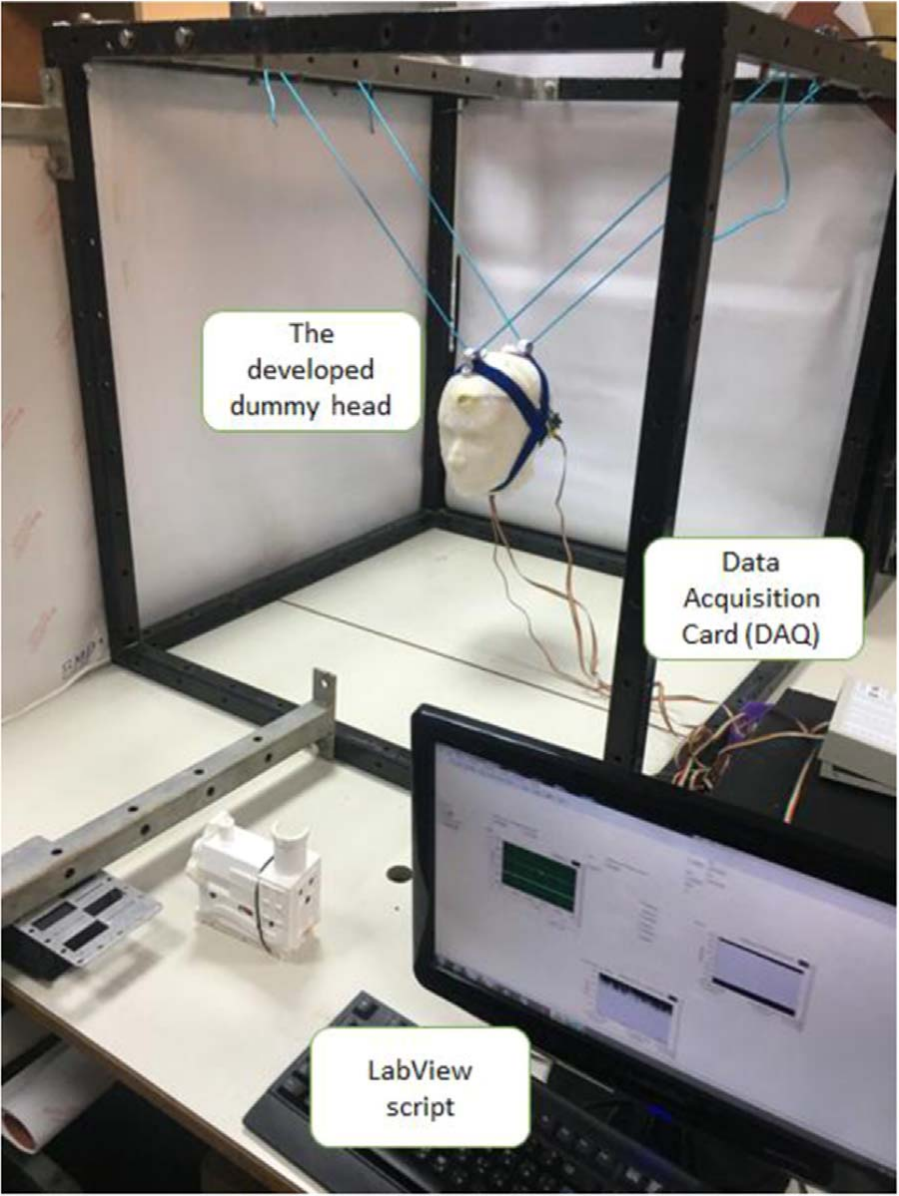
The experimental setup used in this study.

The low-cost head model was situated in a dedicated frame (Fig. 1). The dimensions of the frame used were (94 × 94 × 94 cm^3^). Nylon coated wire ropes were used to position the head at the center of the frame. The accelerometer sensor was interfaced to a computer through a data acquisition card (PCI-6031E, National Instrument, USA) at a sampling rate of 20 kHz and then filtered according to Channel Frequency Class 60. More details about the experimental setup can be found in [3].

### 2.2 Impactors

**Fig. 2:**
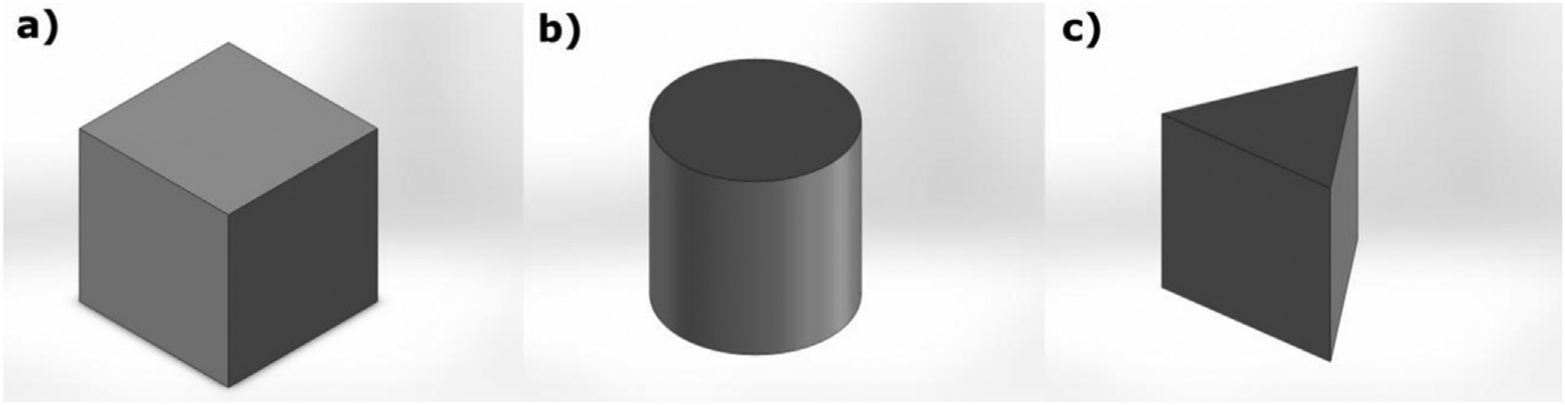
The three shapes that have been used in the experiments. a) Cube. b) Cylinder. c) Wedge.

3D models representing three different basic shapes were developed (Fig. 2). The dimensions of the shapes were (10 × 10 × 10 cm^3^, length, width and height) for the cube, (10 × 10 cm^2^, diameter and height) for the cylinder, and (10 × 10 cm^2^, length and height) for the equilateral triangle base wedge. Dimensions selected are believed to be close to that of a small robot [3], [4], [5]. These were constructed using a 3D printer (Replicator 5th Generation, MakerBot Industries, USA). The volume of each shape was filled with clay to meet the three different mass levels (i.e. 0.3 kg, 0.4 kg, and 0.5 kg). The clay material was placed in such a way to keep the center of mass consistent and balanced.

### 2.3 Procedures

**Fig. 3:**
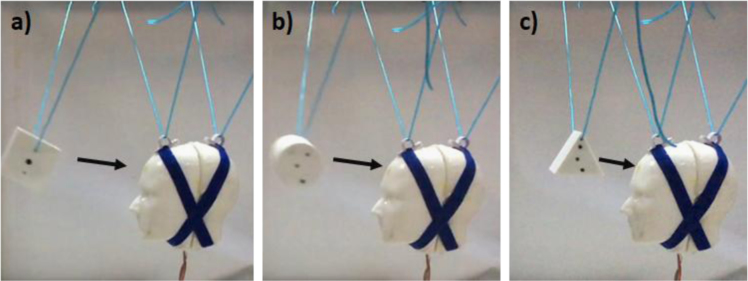
Some of the experiments that have been conducted in this study a) Sample of the experiments with the cube shape. b) Sample of the experiments with the cylinder shape. c) Sample of the experiments with the wedge shape.

To achieve more consistency in terms of impact velocities levels, all experiments were conducted in a controlled condition by tiding the objects to the frame and allow it to swing freely resembling pendulum mechanism (Fig. 3). This method enabled the generation of different impact velocities by altering the drop height of the objects. Four different drop locations generated four different impact velocities levels. The impact velocities based on video analysis were in the range of 1–3 m/s.

All experiments were recorded using a video camera (FDR-X1000V, Sony, Japan) in slow-motion mode (240 fps, 720 pixels). All videos were analyzed to measure the impact velocities using an open-source video analysis software (Tracker version 4.10.0, Douglas Brown, Open Source Physics). A LabView (2014, National Instrument, USA) script was used to obtain the raw readings from the data acquisition card, processes it and then stores it in a worksheet file. The data were post-processed by a Matlab (Version 2015, MathWorks, Massachusetts, USA) script that computes for the peak acceleration value, 3 ms criterion, and the Head Injury criterion (HIC).

## Footnotes

1 To open the TDMS files in Microsoft Excel, you need to install the following Add-In for Microsoft Excel from National Instruments: http://www.ni.com/example/27944/en/.
